# Supplementary material for: Robust Distribution‐Free Tests for the Linear Model
Source: Stat Med. 2026 Feb 5;45(3-5):e70404. doi: 10.1002/sim.70404 (PMC12875190; doi:10.1002/sim.70404)
Supplement: Supplementary file 1 — Data S1. sim70404‐sup‐0001‐Supinfo.pdf. [file SIM-45-0-s001.pdf]

□

APPENDIX

A ADDITIONAL DETAILS FOR HUBER REGRESSION

Algorithm A1 Huber regression with MAD scale estimation[1]

Require: Rectangular data set $[Y, C]$. $Y \in \mathbb{R}^n$ are responses; $C \in \mathbb{R}^{n\times r}$ are covariates.
  $R^0 \leftarrow$ Residuals from OLS regression of Y on C.
  while Not converged **do**
    $s^k \leftarrow 1.4826 * \texttt{median}_{i=1,\ldots,n}(\texttt{abs}(R_i^k))$
    for $i = 1, \ldots, n$ **do**
      $w_i^k \leftarrow \min\left(1, \frac{1.345}{\texttt{abs}(R_i^k)/s^k}\right)$
    end for
    $R^{k+1} \leftarrow$ Residuals from WLS regression of R^k on C with weights w^k.
    Terminate if $\frac{\|R^{k+1}-R^k\|_2}{\|R^k\|_2} <$ Threshold
  end while
  return $R^{\texttt{Final}}$, $s^{\texttt{Final}}$

Algorithm A2 Huber regression with fixed scale[1]

Require: $Y \in \mathbb{R}^n$ are responses; $C \in \mathbb{R}^{n\times r}$ are covariates; $s \in \mathbb{R}$, $s > 0$ is a fixed scale.
  $R^0 \leftarrow$ Residuals from OLS regression of Y on C.
  while Not converged **do**
    for $i = 1, \ldots, n$ **do**
      $w_i^k \leftarrow \min\left(1, \frac{1.345}{\texttt{abs}(R_i^k)/s}\right)$
    end for
    $R^{k+1} \leftarrow$ Residuals from WLS regression of R^k on C with weights w^k.
    Terminate if $\frac{\|R^{k+1}-R^k\|_2}{\|R^k\|_2} <$ Threshold
  end while
  return $R^{\texttt{Final}}$, $s^{\texttt{Final}}$

Our suggested Huber regression based approach relies on the ability to perform scale estimation in a way that satisfies conditions 1 and 2 of section 3 of the main paper. In algorithm A1, we provide an example of such an algorithm, which we note is the default method used for a Huber regression in the MASS package for R[1]. We prove two properties for the output of algorithm A1; analogous properties hold for algorithm A2 with essentially the same proof.

Let $H(C)$ be the "hat matrix" (or projection matrix) that projects a vector Y onto the column space of C. Let Π_π denote the permutation matrix for a permutation $\pi \in S_n$.

Lemma 1. *Let $R_{Huber}(Y; C), s_{Huber}(Y; C)$ be the output of algorithm A1 with covariates C. Then for any $\gamma \in \mathbb{R}^r$, we have*

$$R_{Huber}(Y + C\gamma; C) = R_{Huber}(Y; C),$$
$$s_{Huber}(Y + C\gamma; C) = s_{Huber}(Y; C),$$

and for any $\pi \in S_n$, we have

$$R_{Huber}(Y_\pi; C) = R_{Huber}(Y; C_{\pi^{-1}})_\pi,$$
$$s_{Huber}(Y_\pi; C) = s_{Huber}(Y; C_{\pi^{-1}}).$$

Proof. For the shift invariance, notice that

$$R^0(Y + C\gamma; C) = Y + C\gamma - H(C)(Y + C\gamma) = Y - H(C)Y + C\gamma - C\gamma = R^0(Y; C).$$

Since all remaining steps of the algorithm depend on Y (or $Y + C\gamma$) only through R^0, the result follows.

Let $\pi \in S_n$. Note that

$$R^0(Y_\pi; C) = Y_\pi - H(C)Y_\pi = \Pi_\pi(Y - H(C_{\pi^{-1}})Y) = R^0(Y; C_{\pi^{-1}})_\pi.$$

Suppose that $R^k(Y_\pi; C) = R^k(Y; C_{\pi^{-1}})_\pi$. Then, since s^k is a symmetric function of R^k, we have that $s^k(Y_\pi; C) = s^k(Y; C_{\pi^{-1}})$. Furthermore, the vector w^k is symmetric in permutations of R^k and s^k, and so $w^k(Y_\pi; C) = w^k(Y; C_{\pi^{-1}})_\pi$. Letting $H_w(C) = I - C(C^T WC)^{-1}C^T W$, we have that

$$\begin{aligned} R^{k+1}(Y_\pi; C) &= R^k(Y_\pi; C) - H_{w^k(Y_\pi;C)}(C)R^k(Y_\pi; C) \\ &= \Pi_\pi\left(R^k(Y; C_{\pi^{-1}}) - H_{w^k(Y;C_{\pi^{-1}})}(C_{\pi^{-1}})R^k(Y; C_{\pi^{-1}})\right) \\ &= R^{k+1}(Y; C_{\pi^{-1}})_\pi. \end{aligned}$$

Hence by induction, since $R^0(Y_\pi; C) = R^0(Y; C_{\pi^{-1}})_\pi$, we have that $R^k(Y_\pi; C) = R^k(Y; C_{\pi^{-1}})_\pi$ for all $k \geq 0$. In particular, $R^{\text{Final}}(Y_\pi; C) = R^{\text{Final}}(Y; C_{\pi^{-1}})_\pi$ and $s^{\text{Final}}(Y_\pi; C) = s^{\text{Final}}(Y; C_{\pi^{-1}})$, as desired. □

To show that the output $\mathfrak{p}_{\text{value}}$ from algorithm 1 controls type I error rate, we need to show that

$$\mathcal{M}(Y, X, [Z, Z_\pi]) = \left(\hat{s}(Y, X, [Z, Z_\pi]), \mathbf{r}(Y, X, [Z, Z_\pi])\right)$$

satisfies conditions 1 and 2. In particular,

$$\begin{aligned} \hat{s}(Y + [Z, Z_\pi]\gamma, X, [Z, Z_\pi]) &= s_{\text{Huber}}(Y + [Z, Z_\pi]\gamma; [Z, Z_\pi]) \\ &= s_{\text{Huber}}(Y; [Z, Z_\pi]) = \hat{s}(Y, X, [Z, Z_\pi]), \\ \mathbf{r}(Y + [Z, Z_\pi]\gamma, X, [Z, Z_\pi]) &= \text{Sort}(R_{\text{Huber}}(Y + [Z, Z_\pi]\gamma; [X, Z, Z_\pi])) \\ &= \text{Sort}(R_{\text{Huber}}(Y; [X, Z, Z_\pi])) = \mathbf{r}(Y, X, [Z, Z_\pi]), \\ \hat{s}(Y_\sigma, X_\sigma, [Z, Z_\pi]_\sigma) &= s_{\text{Huber}}(Y_\sigma; [Z, Z_\pi]_\sigma) \\ &= s_{\text{Huber}}(Y; [Z, Z_\pi]) = \hat{s}(Y, X, [Z, Z_\pi]), \\ \mathbf{r}(Y_\sigma, X_\sigma, [Z, Z_\pi]_\sigma) &= \text{Sort}(R_{\text{Huber}}(Y_\sigma, [X, Z, Z_\pi]_\sigma)) \\ &= \text{Sort}(R_{\text{Huber}}(Y; [X, Z, Z_\pi])) = \mathbf{r}(Y, X, [Z, Z_\pi]). \end{aligned}$$

Hence we can apply theorem 1.

B ADDITIONAL DETAILS FOR THE DESIGN OF SIMULATIONS

We run two main experiments. One experiment aims to find the effect that the error distribution, covariate distribution, and number of covariates has on the power of the methods; we will refer to this as the factorial experiment. The other experiment aims to find the effect that the error distribution, covariate distribution, and number of samples has on the power of the methods; we will refer to this as the sample size experiment.

For both experiments, we first generate the covariates $[X, Z]$ and ϵ, and then set

$$Y = X\beta + Z\theta + \epsilon.$$

We use $\theta = 0$ in all simulations. Since all tested methods eliminate the effect of $Z\theta$, this has no effect on the results. We select β such that the F-test at nominal level $\alpha = 0.05$ for the partial correlation of X with Y has a particular power, ranging from $\{0.2, 0.4, 0.6, 0.8, 0.95\}$. To do this, we generate 40000 versions of X, Z, and ϵ to get a Monte-Carlo estimate for the power of the F-test for each choice of β. We then use the Brentq root finding approach, implemented in Scipy[2] to find β such that the Monte-Carlo evaluated F-test power matches the target. After selecting β, for both experiments we use 1000 trials in each setting and $B = 999$ Monte-Carlo replicates for the p-values.

For selecting the covariates, $[X, Z]$, we use random designs with varying tail weights. The tail weights allow us to understand the effect that the distribution of leverages has on the test. In particular, designs that use heavier tailed covariates will generally have more skewed distributions of the individual case leverages. All settings include an intercept column in Z, which counts as one of the dimensions of p (i.e. in the $p = 2$ setting, Z has one intercept column and one additional column). We consider the following choices for $[X, Z]$:

- $[X, Z] \overset{\text{iid}}{\sim} N(0, 1)$ - the Normal design.
- $[X, Z] \overset{\text{iid}}{\sim} t_3$ - the t_3 design.
- $[X, Z] \overset{\text{iid}}{\sim} \text{Cauchy}(0, 1)$ - the Cauchy design.
- $[X, Z]$ is all $\{0, 1\}$ valued, with each row containing only an intercept and one treatment - the Balanced ANOVA design.

In the Balanced ANOVA design, the sample size n is rounded down to the nearest value such that there are the same number of observations for each treatment. We found that the empirical distribution of the leverages has approximately the same effect on the F-test as it has on RobustPALMRT's performance, and so in the main body of the paper we only reported results in the Normal design setting.

For selecting the errors, ϵ, we wanted to assess how tail weight and skewness affect the various methods. We consider the following choices for ϵ:

- $\epsilon \overset{\text{iid}}{\sim} N(0, 1)$ - Normal errors.
- $\epsilon \overset{\text{iid}}{\sim} t_3$ - t_3 errors.
- $\epsilon \overset{\text{iid}}{\sim} \text{Cauchy}(0, 1)$ - Cauchy errors.
- $\epsilon \overset{\text{iid}}{\sim} \text{LogNormal}(\sigma = 1)$ - Log Normal errors.
- $\epsilon \sim N(0, I_n) + (-1)^{\text{Bernoulli}(0.5)} \cdot 10^4 \cdot \text{Multinomial}\left(1; \frac{1}{n}, \ldots, \frac{1}{n}\right)$ - Multinomial errors.

The Log Normal setting has individual entries $\log(\epsilon_i) \sim N(0, 1)$. This is a moderately skewed setting, having skewness $\gamma_1 = 6.18$. The Multinomial errors setting represents a Normal errors setting except with one extreme outlier.

Lastly, for each setting, we additionally evaluated the type I error rate by setting $\beta = 0$ and otherwise following the same procedures.

In the factorial experiment, we vary $p \in \{2, 6, 16\}$, and we test all 20 design/error distribution combinations.

In the sample size experiment, we fix $p = 6$, and we test the most interesting settings from the factorial setting, varying $n \in \{25, 50, 100, 200, 400\}$. The selected settings were

- Normal design, Normal errors.
- Normal design, t_3 errors.
- Normal design, Cauchy errors.
- Normal design, Log Normal errors.
- Normal design, Multinomial errors.
- Cauchy design, Normal errors.
- Cauchy design, t_3 errors.
- Cauchy design, Cauchy errors.
- Cauchy design, Log Normal errors.
- Cauchy design, Multinomial errors.
- t_3 design, Log Normal errors.
- Balanced ANOVA design, Log Normal errors.

This collection of settings includes all of the error distributions we consider along with both normal and Cauchy errors. The Normal designs tend to have no or only a couple high leverage points, whereas the Cauchy design creates a few very high leverage cases. Additionally we include two more settings with Log Normal errors in a variety of settings, since the skewed errors setting is a large part of the novelty of these methods. However, we did not find any surprising results in this direction, and so for the sake of ease of presentation we plotted results only for the Normal and Cauchy designs.

All of the experiments were run using a cluster with 40 CPUs @ 2.40 GHz. The total runtime across all experiments was 109 hours. Originally we ran the experiments with a fixed scale rather than using scale estimation; the results from those experiments did not make it into the paper, since the estimated scale setting is more realistic and interesting. Code is available to reproduce all of the results, or to efficiently produce similar results in new settings. We select seeds in an arithmetic progression, and for reproducibility our outputs include which seed was used. On a personal computer, it is feasible to assess the power of these methods in a new setting (for example, a different design, sample size, or error distribution) that is similar to the tested ones in under an hour of compute time.

C PROOF OF THEOREM 1

Our proof of theorem 1 owes much to the development in Guan.[3] We take a slightly different approach, however, in defining a more general *population* p-value and showing that it controls type I error. We then use this to derive the guarantee for a Monte-Carlo p-value that relies on the randomly selected permutations $\pi_1, \ldots, \pi_B$. This approach follows the ideas in Ramdas et al.[4] for creating permutation tests with permutations drawn from some non-uniform distribution of permutations F over the group of permutations S_n.

For the remainder of this section, let us assume that $H_0 : \beta = 0$ holds; that is, $Y = Z\theta + \epsilon$ for some fixed $\theta \in \mathbb{R}^p$ and some exchangeable distribution for ϵ. Furthermore, assume that we have a model fitting procedure $\mathcal{M}$ that satisfies conditions 1 and 2. Our proof applies to a more general setting, where the model evaluation procedure is allowed to simultaneously consider information from both model fits that it is comparing. We formalize this by defining a model comparison function $\tilde{\omega}(M_1, M_2) \in [0, 1]$ that satisfies $\tilde{\omega}(M_1, M_2) = 1 - \tilde{\omega}(M_2, M_1)$. The framework discussed in the paper uses

$$\tilde{\omega}(M_1, M_2) = I(\omega(M_1) > \omega(M_2)) + \frac{1}{2} I(\omega(M_1) = \omega(M_2)).$$

Notice that this choice handles ties in a slightly different fashion than is done in the main paper. For ease of presentation, in the main paper we handled ties conservatively and thus the p-values in the main paper are greater than the p-values discussed here, so the theory will carry over.

Recall that for each $\pi \in S_n$, we compute

$$\begin{aligned} M^{\pi}_{\text{Orig}} &= \mathcal{M}(Y, X, [Z, Z_\pi]) \\ &\overset{H_0}{=} \mathcal{M}(Z\theta + \epsilon, X, [Z, Z_\pi]) \\ &= \mathcal{M}(\epsilon, X, [Z, Z_\pi]), \\ M^{\pi}_{\text{Perm}} &= \mathcal{M}(Y, X_\pi, [Z, Z_\pi]) \\ &\overset{H_0}{=} \mathcal{M}(Z\theta + \epsilon, X_\pi, [Z, Z_\pi]) \\ &= \mathcal{M}(\epsilon, X_\pi, [Z, Z_\pi]). \end{aligned}$$

Hence under the null hypothesis, the expression $\omega(M^{\pi}_{\text{Orig}}, M^{\pi}_{\text{Perm}})$ does not depend on the unknown effect of the control covariates, θ. For any pair of permutations $\pi, \sigma \in S_n$, the exchangeability of ϵ implies that

$$\begin{aligned} \tilde{\omega}(M^{\pi}_{\text{Orig}}, M^{\pi}_{\text{Perm}}) &\overset{H_0}{=} \tilde{\omega}(\mathcal{M}(\epsilon, X, [Z, Z_\pi]), \mathcal{M}(\epsilon, X_\pi, [Z, Z_\pi])) \\ &\overset{d}{=} \tilde{\omega}(\mathcal{M}(\epsilon_{\sigma^{-1}}, X, [Z, Z_\pi]), \mathcal{M}(\epsilon_{\sigma^{-1}}, X_\pi, [Z, Z_\pi])) \\ &= \tilde{\omega}(\mathcal{M}(\epsilon, X_\sigma, [Z_\sigma, Z_{\sigma\circ\pi}]), \mathcal{M}(\epsilon, X_{\sigma\circ\pi}, [Z_\sigma, Z_{\sigma\circ\pi}])) \\ &= \tilde{\omega}(\mathcal{M}(\epsilon, X_\sigma, [Z_\sigma, Z_\tau]), \mathcal{M}(\epsilon, X_\tau, [Z_\sigma, Z_\tau])), \end{aligned}$$

where we reparameterized to $\tau = \sigma \circ \pi$. This suggests that we define the array $A \in \mathbb{R}^{n! \times n!}$ by

$$A(\tau, \sigma; \epsilon) = \tilde{\omega}(\mathcal{M}(\epsilon, X_\sigma, [Z_\sigma, Z_\tau]), \mathcal{M}(\epsilon, X_\tau, [Z_\sigma, Z_\tau])). \tag{C1}$$

Notice that the p-value (7) satisfies the following:

$$\begin{aligned}
\mathfrak{p}_{\text{value}} &= \frac{1 + \sum_{b=1}^{B} \tilde{\omega}(M_{\text{Orig}}^{\pi_b}, M_{\text{Perm}}^{\pi_b})}{1+B} \\
&\overset{H_0}{=} \frac{1 + \sum_{b=1}^{B} \tilde{\omega}(\mathcal{M}(\epsilon, X, [Z, Z_{\pi_b}]), \mathcal{M}(\epsilon, X_{\pi_b}, [Z, Z_{\pi_b}]))}{1+B} \\
&= \frac{1 + \sum_{b=1}^{B} A(\pi_b, Id; \epsilon)}{1+B} \\
&\overset{d}{=} \frac{1 + \sum_{b=1}^{B} A(\pi_b, Id; \epsilon_{\sigma^{-1}})}{1+B} \\
&= \frac{1 + \sum_{b=1}^{B} A(\sigma \circ \pi_b, \sigma; \epsilon)}{1+B} \\
&= \frac{1}{1+B} + \sum_{b=1}^{B} \frac{1}{1+B} A(\sigma \circ \pi_b, \sigma; \epsilon) \\
&\overset{B\to\infty}{\longrightarrow} \sum_{\pi \in S_n} \mathbb{P}_{\pi_b}(\pi_b = \pi) A(\sigma \circ \pi, \sigma; \epsilon) \\
&= \mathbb{E}_{\pi_b}[A(\sigma \circ \pi_b, \sigma; \epsilon)|\epsilon].
\end{aligned}$$

We have not yet appealed to any properties of the randomly selected π_b. Let $\pi_b \sim F$, for some distribution F on the group of permutations S_n, with probability mass function $f(\pi)$. If F is a uniform distribution on some subgroup G of S_n, then $\sigma \circ \pi_b \overset{d}{=} \pi_b$, and so

$$\begin{aligned}
\mathbb{E}_{\pi_b}\left[A(\pi_b, Id; \epsilon) \mid \epsilon\right] &\overset{d}{=} \mathbb{E}_{\pi_b}\left[A(\pi_b, Id; \epsilon_{\sigma^{-1}}) \mid \epsilon\right] \\
&= \mathbb{E}_{\pi_b}\left[A(\sigma \circ \pi_b, \sigma; \epsilon) \mid \epsilon\right] \\
&= \mathbb{E}_{\pi_b}\left[A(\pi_b, \sigma; \epsilon) \mid \epsilon\right].
\end{aligned}$$

Since $\mathbb{E}_{\pi_b}\left[A(\pi_b, \tau; \epsilon)|\epsilon\right]$ is the average of column τ of the matrix A, and $A(\pi_b, \tau; \epsilon) + A(\tau, \pi_b; \epsilon) = 1$, we are prompted to bound the probability that the average of a *randomly* selected column is particularly small.

We now have sufficient motivation to formally state our result. Let F be a (possibly non-uniform) distribution on the group of permutations S_n, with probability mass function $f(\pi)$. Select $\sigma \overset{ind}{\sim} F$, and define the *population* p-value as:

$$\mathfrak{p}_{\text{value}}^{\text{Pop}}(\sigma) = \mathbb{E}_{\pi_b \sim F}\left[A(\sigma^{-1} \circ \pi_b, Id; \epsilon) \mid \epsilon\right] \tag{C2}$$

$$\overset{H_0}{=} \mathbb{E}_{\pi_b \sim F}\left[A(\sigma^{-1} \circ \pi_b, Id; Y) \mid \epsilon\right] \tag{C3}$$

Furthermore, if we select $\sigma, \pi_1, \ldots, \pi_B \overset{\text{iid}}{\sim} F$, then we define the *Monte-Carlo* p-value as:

$$\mathfrak{p}_{\text{value}}^{\text{MC}}(\sigma) = \frac{1 + \sum_{b=1}^{B} A(\sigma^{-1} \circ \pi_b, Id; \epsilon)}{1+B} \tag{C4}$$

$$\overset{H_0}{=} \frac{1 + \sum_{b=1}^{B} A(\sigma^{-1} \circ \pi_b, Id; Y)}{1+B}]. \tag{C5}$$

Then we have the following result:

Theorem 1. *Suppose $\mathcal{M}$ satisfies conditions 1, 2 and $\tilde{\omega}$ satisfies $\tilde{\omega}(M_1, M_2) + \tilde{\omega}(M_2, M_2) = 1$. Let Y be generated according to model* (3)*, and let $\sigma, \pi_1, \ldots, \pi_B \overset{\text{iid}}{\sim} F$ for any (possibly non-uniform) distribution F over the group of permutations S_n. Then for*

any $\theta \in \mathbb{R}^p$ and any exchangeable ϵ, under the null hypothesis H_0 we have:

$$\mathbb{P}_{H_0}\left(\mathfrak{p}_{\text{value}}^{\text{Pop}}(\sigma) \leq \alpha\right) \leq 2\alpha,$$
$$\mathbb{P}_{H_0}\left(\mathfrak{p}_{\text{value}}^{\text{MC}}(\sigma) \leq \alpha\right) \leq 2\alpha,$$

where the randomness is averaged over ϵ, σ, and $\pi_1, \ldots, \pi_B$.

If, additionally, F is uniform distribution over some subgroup $G \subseteq S_n$, then

$$\mathfrak{p}_{\text{value}}^{\text{Pop}}(\sigma) \overset{d}{=} \mathfrak{p}_{\text{value}}^{\text{Pop}}(Id),$$
$$\mathfrak{p}_{\text{value}}^{\text{MC}}(\sigma) \overset{d}{=} \mathfrak{p}_{\text{value}}^{\text{MC}}(Id),$$

and so we have

$$\mathbb{P}_{H_0}\left(\mathfrak{p}_{\text{value}}^{\text{Pop}}(Id) \leq \alpha\right) \leq 2\alpha,$$
$$\mathbb{P}_{H_0}\left(\mathfrak{p}_{\text{value}}^{\text{MC}}(Id) \leq \alpha\right) \leq 2\alpha,$$

where the randomness includes only ϵ and $\pi_1, \ldots, \pi_B$.

Theorem 1 is a special case of this result, because the p-value (7) in the main text is $\mathfrak{p}_{\text{value}}^{\text{MC}}(Id)$ and F is the uniform distribution on S_n. In the case of a uniform distribution on a subgroup, we do not need to generate the random permutation $\sigma \sim F$, and so the only noise in our p-value is the Monte-Carlo noise due to $\{\pi_1, \ldots, \pi_B\}$; this is relatively benign, since the p-value will converge to the non-randomized *population* p-value $\mathfrak{p}_{\text{value}}^{\text{Pop}}(Id)$ as B tends to infinity. If F is non-uniform, then the test is genuinely a randomized test, which may be undesirable. We note that to reduce the variance due to this randomization, one can sample many choices for $\sigma \sim F$ and average the corresponding p-values at the cost of an additional factor of 2 in the type I error control guarantee.[5] More precisely, we can sample $\sigma_1, \ldots, \sigma_M \overset{\text{iid}}{\sim} F$ and get

$$\mathbb{P}_{H_0}\left(\frac{1}{M}\sum_{j=1}^{M} \mathfrak{p}_{\text{value}}^{\text{Pop}}(\sigma_j) \leq \alpha\right) \leq 4\alpha.$$

See Ramdas et al.[4] for more discussion on this. It is unclear whether the benefit of selecting a non-uniform distribution for F outweighs the cost of a substantially worse bound on the type I error.

Proof. For notational clarity, we write subscripts on $\mathbb{P}$ to denote which variables are random in the given expressions. We first control the population type I error rate:

$$\begin{aligned}
\mathbb{P}_{\epsilon,\sigma\sim F}\left(\mathfrak{p}^{\text{Pop}}_{\text{value}}(\sigma) \le \alpha\right) &= \mathbb{P}_{\epsilon,\sigma\sim F}\left(\mathbb{E}_{\pi_b\sim F}\left[A(\sigma^{-1}\circ\pi_b, Id;\epsilon) \mid \epsilon\right] \le \alpha\right) \\
&= \sum_{\tau\in S_n} \mathbb{P}_{\epsilon,\sigma\sim F}\left(\sigma = \tau, \mathbb{E}_{\pi_b\sim F}\left[A(\tau^{-1}\circ\pi_b, Id;\epsilon) \mid \epsilon\right] \le \alpha\right) \\
\text{(Indep. of } \sigma \text{ and } \epsilon) &= \sum_{\tau\in S_n} \mathbb{P}_{\sigma\sim F}\left(\sigma = \tau\right)\mathbb{P}_{\epsilon}\left(\mathbb{E}_{\pi_b\sim F}\left[A(\tau^{-1}\circ\pi_b, Id;\epsilon) \mid \epsilon\right] \le \alpha\right) \\
\text{(Symmetry of } \mathcal{M}) &= \sum_{\tau\in S_n} \mathbb{P}_{\sigma\sim F}\left(\sigma = \tau\right)\mathbb{P}_{\epsilon}\left(\mathbb{E}_{\pi_b\sim F}\left[A(\pi_b, \tau;\epsilon_\tau) \mid \epsilon\right] \le \alpha\right) \\
\text{(Exch. of } \epsilon) &= \sum_{\tau\in S_n} \mathbb{P}_{\sigma\sim F}\left(\sigma = \tau\right)\mathbb{P}_{\epsilon}\left(\mathbb{E}_{\pi_b\sim F}\left[A(\pi_b, \tau;\epsilon) \mid \epsilon\right] \le \alpha\right) \\
&= \sum_{\tau\in S_n} f(\tau)\mathbb{E}_{\epsilon}\left[I\left(\sum_{\pi\in S_n} f(\pi)A(\pi,\tau;\epsilon) \le \alpha\right)\right] \\
&= \mathbb{E}_{\epsilon}\left[\sum_{\tau\in S_n} f(\tau)I\left(\sum_{\pi\in S_n} f(\pi)A(\pi,\tau;\epsilon) \le \alpha\right)\right] \\
\text{(Lemma 2)} &\le \mathbb{E}_{\epsilon}[2\alpha] \\
&= 2\alpha.
\end{aligned}$$

As an aside, note that the upcoming lemma 2 provides a deterministic bound on the amount of weight that can be placed on columns with particularly small weighted column sum, in any array A_{ij} with entries in $[0,1]$ that satisfy $A_{ij}+A_{ji} = 1$. While it is tempting to try to directly appeal to column exchangeability properties, only the values in the column $A(\pi, Id;\epsilon) = A(\pi; Id;, Y)$ will be computable from the data. In order to compute $A(\pi,\sigma;\epsilon)$ for some $\sigma \neq Id$, we would need to know the true value of ϵ. Nonetheless, the $A_{ij} + A_{ji} = 1$ condition lets us bound how many columns can have small column sums, and we can use the symmetry of the matrix $A(\pi,\sigma;\epsilon)$ to relate properties of a randomly selected column to properties of our particular p-value.

Continuing the proof in the style of Ramdas et al.,[4] we now control the type I error for the Monte-Carlo version. Let $\sigma, \pi_1, \ldots, \pi_B \overset{\text{iid}}{\sim} F$, and for ease of notation let $\pi_0 = \sigma$. Notice that the vector $(\pi_0, \pi_1, \ldots, \pi_B)$ is exchangeable, and thus for any $\tau \in S_{B+1}$, a permutation of the set $\{0, \ldots, B\}$, conditioned on the multiset $\{\pi_0, \ldots, \pi_B\}$ we have

$$(\pi_{\tau(0)}, \pi_{\tau(1)}, \ldots, \pi_{\tau(B)}) \overset{d}{=} (\pi_0, \pi_1, \ldots, \pi_B).$$

In particular, notice that if we define, for any $\tilde{\sigma}, \tilde{\pi}_0, \ldots, \tilde{\pi}_B$,

$$g(\tilde{\sigma}, \tilde{\pi}_0, \ldots, \tilde{\pi}_B) = \frac{\sum_{b=0}^{B} A(\tilde{\sigma}^{-1}\circ\tilde{\pi}_b, Id;\epsilon)}{1+B},$$

then we have (conditioned on $\{\pi_0, \ldots, \pi_B\}$):

$$g(\pi_{\tau(0)}, \pi_{\tau(0)}, \ldots, \pi_{\tau(B)}) \overset{d}{=} g(\pi_0, \pi_0, \ldots, \pi_B). \tag{C6}$$

Furthermore, for any $\tau \in S_{B+1}$, we have

$$\begin{aligned}
g(\tilde{\sigma}, \tilde{\pi}_{\tau(0)}, \ldots, \tilde{\pi}_{\tau(B)}) &= \frac{\sum_{b=0}^{B} A(\tilde{\sigma}^{-1}\circ\tilde{\pi}_{\tau(b)}, Id;\epsilon)}{1+B} \\
&= \frac{\sum_{b=0}^{B} A(\tilde{\sigma}^{-1}\circ\tilde{\pi}_b, Id;\epsilon)}{1+B} \\
&= g(\tilde{\sigma}, \tilde{\pi}_0, \ldots, \tilde{\pi}_B).
\end{aligned} \tag{C7}$$

Equipped with these two facts, we will first describe the p-values in terms of g, and then we will appeal to the validity of the population p-value for *any* choice of F. In particular, define the empirical distribution $\{\pi_0, \dots, \pi_B\}$:

$$\tilde{F} = \frac{1}{B+1}\sum_{b=0}^{B}\delta_{\pi_b}.$$

Then selecting $K \sim \text{Unif}\{0, \dots, B\}$, we have $\pi_K \sim \tilde{F}$. Then by the population validity result,

$$\mathbb{P}_{\epsilon,K,\pi_0,\dots,\pi_B}\left(\mathfrak{p}^{\text{Pop}}_{\text{value}}(\pi_K) \le \alpha \;\middle|\; \{\pi_0, \dots, \pi_B\}\right) \le 2\alpha.$$

Notice that

$$\begin{aligned}
\mathfrak{p}^{\text{Pop}}_{\text{value}}(\pi_K) &= \mathbb{E}_{\tilde{\pi}\sim\tilde{F}}\left[A(\pi_K^{-1}\circ\tilde{\pi}, Id; \epsilon) \mid \epsilon\right] \\
&= \frac{\sum_{b=0}^{B} A(\pi_K^{-1}\circ\pi_b, Id; \epsilon)}{B+1} \\
&= g(\pi_K, \pi_0, \dots, \pi_B),
\end{aligned}$$

and lastly notice that

$$\begin{aligned}
\mathfrak{p}^{\text{MC}}_{\text{value}}(\sigma) &= \mathfrak{p}^{\text{MC}}_{\text{value}}(\pi_0) \\
&= \frac{1 + \sum_{b=1}^{B} A(\pi_0^{-1}\circ\pi_b, Id; \epsilon)}{1+B} \\
&= \frac{\frac{1}{2} + \sum_{b=0}^{B} A(\pi_0^{-1}\circ\pi_b, Id; \epsilon)}{1+B} \\
&= g(\pi_0, \pi_0, \dots, \pi_B) + \frac{1}{2(1+B)}.
\end{aligned}$$

Calling $\tau_k \in S_{B+1}$ the permutation that swaps 0 and k for any $k \in \{0, \dots, B\}$, we have

$$\begin{aligned}
&\mathbb{P}_{\epsilon,K,\pi_0,\dots,\pi_B}\left(\mathfrak{p}^{\text{MC}}_{\text{value}}(\pi_0) \le \alpha \;\middle|\; \{\pi_0, \dots, \pi_B\}\right) \\
&= \mathbb{P}_{\epsilon,K,\pi_0,\dots,\pi_B}\left(g\left(\pi_0, \pi_0, \dots, \pi_B\right) + \frac{1}{2(1+B)} \le \alpha \;\middle|\; \{\pi_0, \dots, \pi_B\}\right) \\
&\le \mathbb{P}_{\epsilon,K,\pi_0,\dots,\pi_B}\left(g\left(\pi_0, \pi_0, \dots, \pi_B\right) \le \alpha \;\middle|\; \{\pi_0, \dots, \pi_B\}\right) \\
\text{(By (C6))} &= \mathbb{P}_{\epsilon,K,\pi_0,\dots,\pi_B}\left(g\left(\pi_{\tau_K(0)}, \pi_{\tau_K(0)}, \dots, \pi_{\tau_K(B)}\right) \le \alpha \;\middle|\; \{\pi_0, \dots, \pi_B\}\right) \\
\text{(By (C7))} &= \mathbb{P}_{\epsilon,K,\pi_0,\dots,\pi_B}\left(g\left(\pi_{\tau_K(0)}, \pi_0, \dots, \pi_B\right) \le \alpha \;\middle|\; \{\pi_0, \dots, \pi_B\}\right) \\
&= \mathbb{P}_{\epsilon,K,\pi_0,\dots,\pi_B}\left(g\left(\pi_K, \pi_0, \dots, \pi_B\right) \le \alpha \;\middle|\; \{\pi_0, \dots, \pi_B\}\right) \\
&= \mathbb{P}_{\epsilon,K,\pi_0,\dots,\pi_B}\left(\mathfrak{p}^{\text{Pop}}_{\text{value}}(\pi_K) \le \alpha \;\middle|\; \{\pi_0, \dots, \pi_B\}\right) \\
&\le 2\alpha.
\end{aligned}$$

Marginalizing over $\{\pi_0, \dots, \pi_B\}$, we have

$$\begin{aligned}
\mathbb{P}_{\epsilon,K,\pi_0,\dots,\pi_B}\left(\mathfrak{p}^{\text{MC}}_{\text{value}}(\pi_0) \le \alpha\right) &= \mathbb{E}_{\epsilon,K,\pi_0,\dots,\pi_B}\left[\mathbb{P}_{\epsilon,K,\pi_0,\dots,\pi_B}\left(\mathfrak{p}^{\text{MC}}_{\text{value}}(\pi_0) \le \alpha \;\middle|\; \{\pi_0, \dots, \pi_B\}\right)\right] \\
&\le E[2\alpha] \\
&= 2\alpha,
\end{aligned}$$

as desired. □

We now present the combinatorial bound on the weighted column sums. This is very similar to a proof in appendix E.4.3 of Barber et al.,[6] but this statement is slightly more general so we reproduce it here:

Lemma 2. *Let $A \in [0,1]^{m\times m}$ be a matrix with the property that*

$$A_{ij} + A_{ji} = 1$$

for all $i, j \in [m]$. Furthermore, let $w_1, \dots, w_m \in [0,1]$ have $\sum_{j=1}^m w_j = 1$. Fix $\alpha \in [0, 1/2]$ and define

$$\begin{aligned} S &= \left\{ i \in [m] \mid \sum_{j=1}^m w_j A_{ij} \geq 1-\alpha \right\} \\ &= \left\{ i \in [m] \mid \sum_{j=1}^m w_j A_{ji} \leq \alpha \right\}. \end{aligned}$$

Then

$$\sum_{i\in[m]} w_i I \left(\sum_{j\in[m]} w_j A_{ji} \leq \alpha \right) = \sum_{i\in S} w_i \leq 2\alpha.$$

Proof. For any $i \in S$,

$$\begin{aligned} 1-\alpha &\leq \sum_{j=1}^m w_j A_{ij} \\ &\leq \sum_{j\in S} w_j A_{ij} + \sum_{j\notin S} w_j \\ &= \sum_{j\in S} w_j A_{ij} + 1 - \sum_{j\in S} w_j. \end{aligned}$$

Then notice that

$$\sum_{i,j\in S} w_i w_j A_{ij} = \sum_{i,j\in S} w_i w_j A_{ji},$$

and so

$$\begin{aligned} \sum_{i,j\in S} w_i w_j A_{ij} &= \frac{1}{2} \sum_{i,j\in S} w_i w_j \left[A_{ij} + A_{ji}\right] \\ &\leq \frac{1}{2} \sum_{i,j\in S} w_i w_j. \end{aligned}$$

Hence

$$\begin{aligned} (1-\alpha) \sum_{i\in S} w_i &\leq \sum_{i\in S} w_i \left[\sum_{j\in S} w_j A_{ij} + 1 - \sum_{j\in S} w_j \right] \\ &= \sum_{i,j\in S} w_i w_j A_{ij} + \sum_{i\in S} w_i - \sum_{i,j\in S} w_i w_i \\ &\leq \frac{1}{2} \sum_{i,j\in S} w_i w_j + \sum_{i\in S} w_i - \sum_{i,j\in S} w_i w_i \\ &= -\frac{1}{2} \sum_{i,j\in S} w_i w_j + \sum_{i\in S} w_i \\ &= -\frac{1}{2} \left(\sum_{i\in S} w_i \right)^2 + \sum_{i\in S} w_i. \end{aligned}$$

Straightforward algebra gives $\sum_{i\in S} w_i \leq 2\alpha$, as desired.

To see that the two versions of S are equal, notice that

$$\sum_{j\in[m]} w_j A_{ji} = \sum_{j\in[m]} w_j(1 - A_{ij})$$
$$= 1 - \sum_{j\in[m]} w_j A_{ij}.$$

□

Corollary 1. *Let $A(\pi, \tau; \epsilon)$ be as* (C1)*. For any distribution F on the group of permutations S_n, with probability mass function $f(\pi)$, we have:*

$$\sum_{\tau\in S_n} f(\tau) I\left(\sum_{\pi\in S_n} f(\pi) A(\pi, \tau; \epsilon) \leq \alpha\right) \leq 2\alpha.$$

Proof. We use lemma 2 with $m = n!$, $A_{\pi,\tau} = A(\pi, \tau; \epsilon)$, and $w_\pi = f(\pi)$. □

D ADDITIONAL PLOTS FOR SIMULATIONS

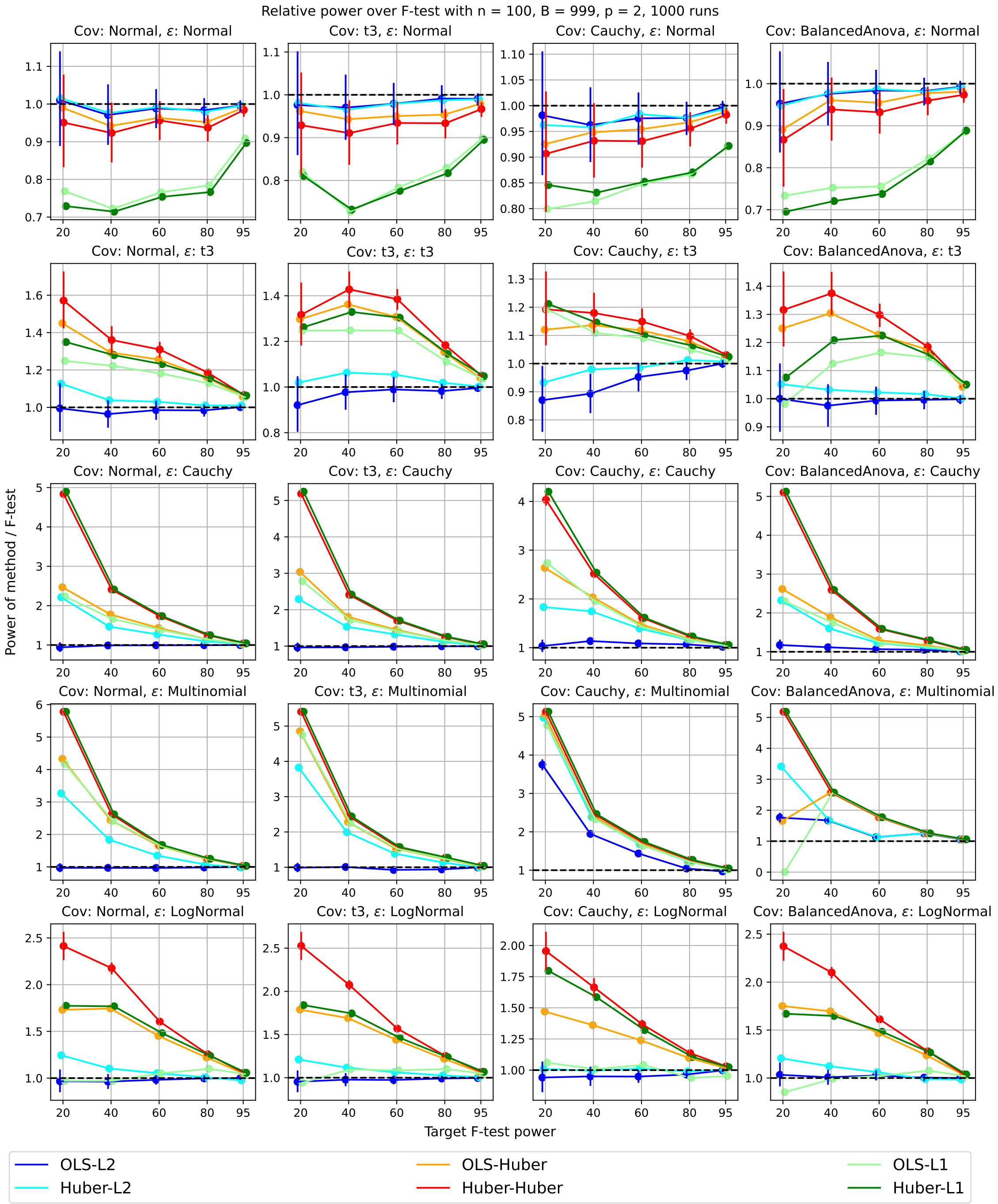

FIGURE D1 Relative power for various RobustPALRMT regression approaches compared to the F-test, computed using 1000 trials. See section 5. Here we use $p = 2$ covariates in Z and sample size of $n = 100$. Monte-Carlo 95% error bars are plotted for the two methods of most interest, that is Huber-Huber RobustPALMRT and OLS-L2 PALMRT. The simulation is blocked, meaning for each setting and target F-test power, in a given replicate, the same data set is used for all methods. This leads to conservative error bars for the differences between the methods.

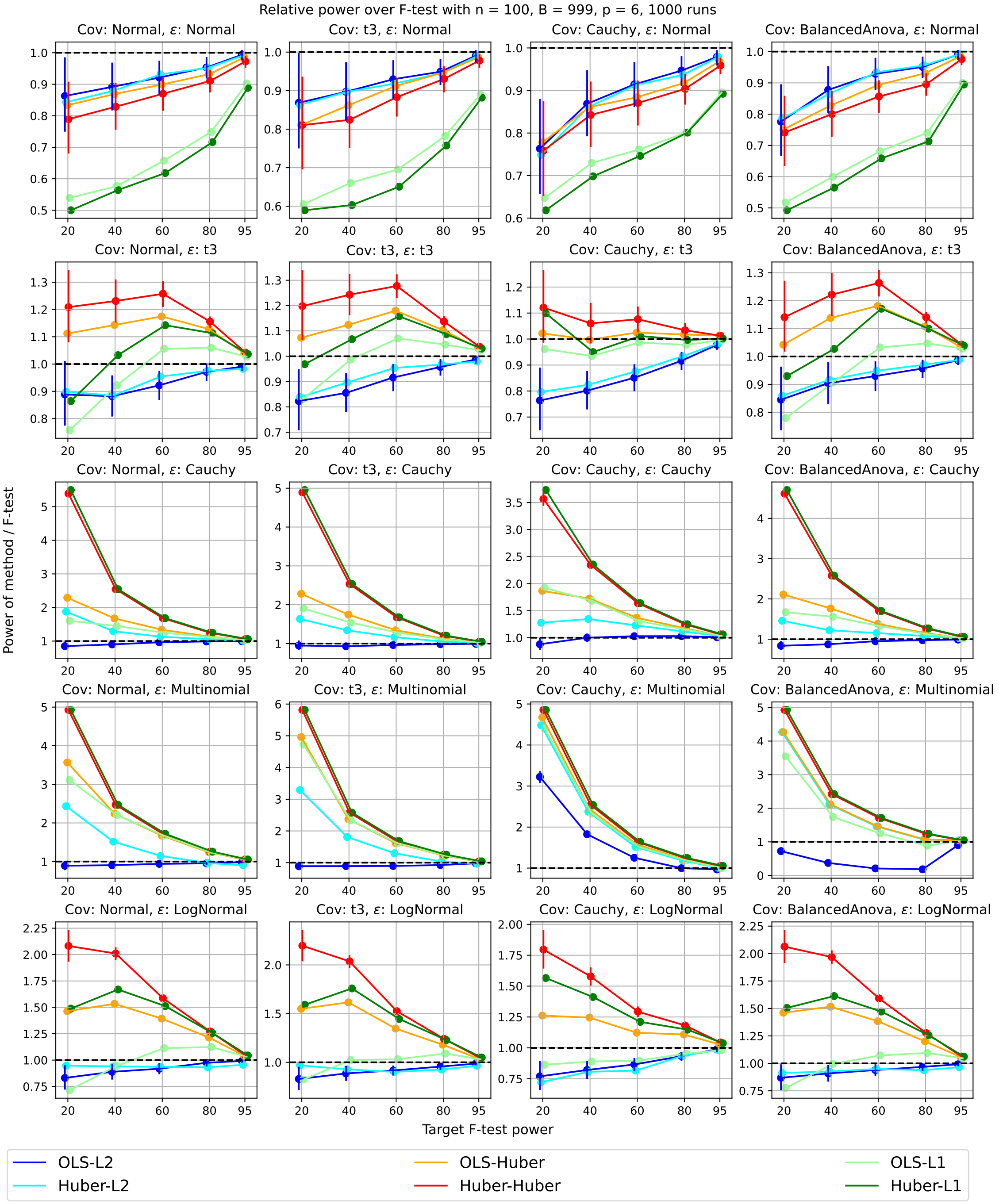

FIGURE D2 Relative power for various RobustPALRMT regression approaches compared to the F-test, computed using 1000 trials. See section 5. Here we use $p = 6$ covariates in Z and sample size of $n = 100$. Monte-Carlo 95% error bars are plotted for the two methods of most interest, that is Huber-Huber RobustPALRMT and OLS-L2 PALMRT. The simulation is blocked, meaning for each setting and target F-test power, in a given replicate, the same data set is used for all methods. This leads to conservative error bars for the differences between the methods.

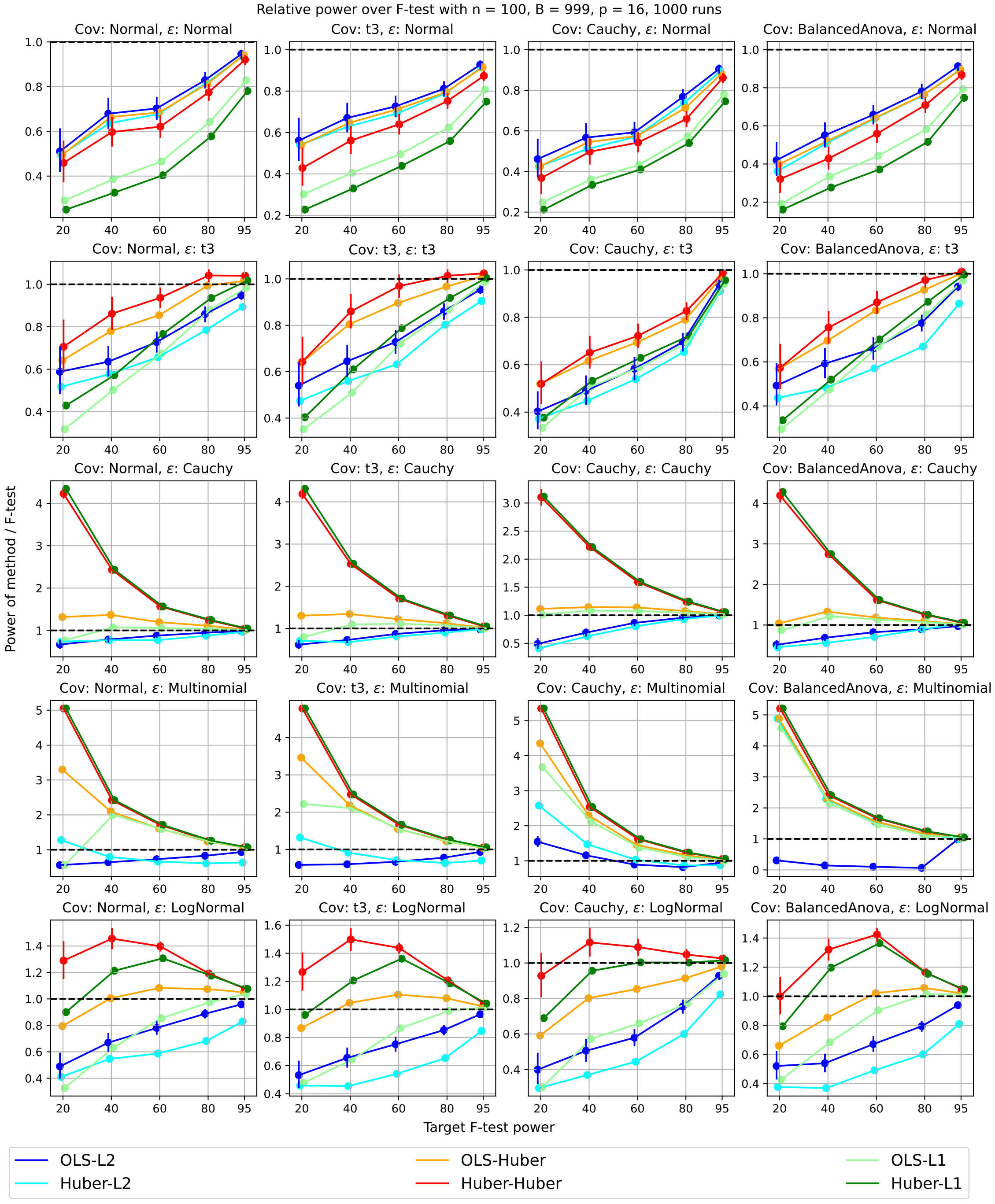

FIGURE D3 Relative power for various RobustPALRMT regression approaches compared to the F-test, computed using 1000 trials. See section 5. Here we use $p = 16$ covariates in Z and sample size of $n = 100$. Monte-Carlo 95% error bars are plotted for the two methods of most interest, that is Huber-Huber RobustPALMRT and OLS-L2 PALMRT. The simulation is blocked, meaning for each setting and target F-test power, in a given replicate, the same data set is used for all methods. This leads to conservative error bars for the differences between the methods.

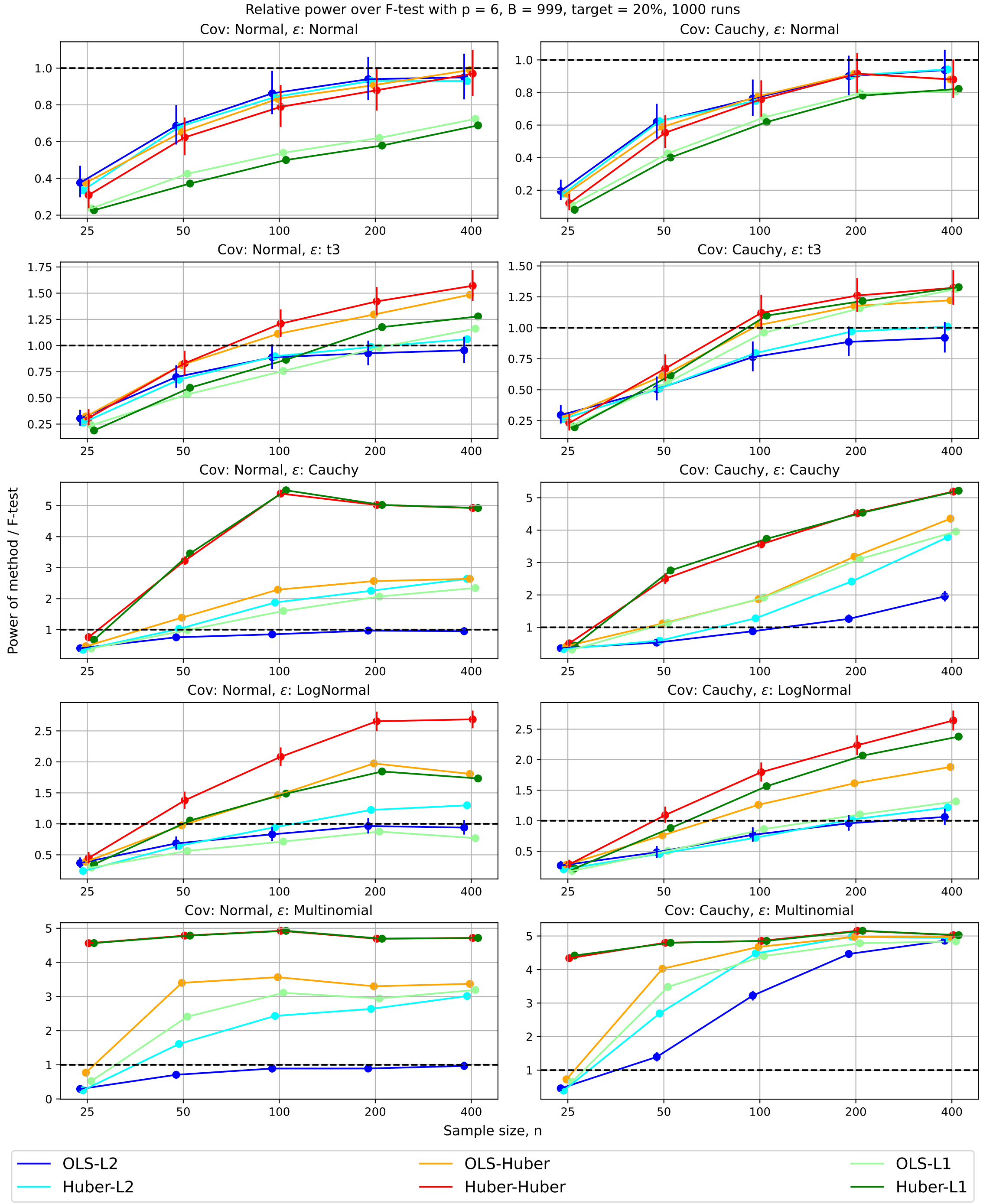

FIGURE D4 Relative power of RobustPALMRT compared to the F-test vs sample size n. In each setting, β is selected to fix the power of the F-test at 20%. Note that the maximum possible ratio of powers is 5. There are 1000 replicates with $B = 999$. We show error bars for our method (Huber-Huber) and (OLS-L2)[3], the two of most interest. To improve the visualization of overlapping curves and error bars, we "jittered" curves horizontally, preserving the ratios and the shapes of the curves.

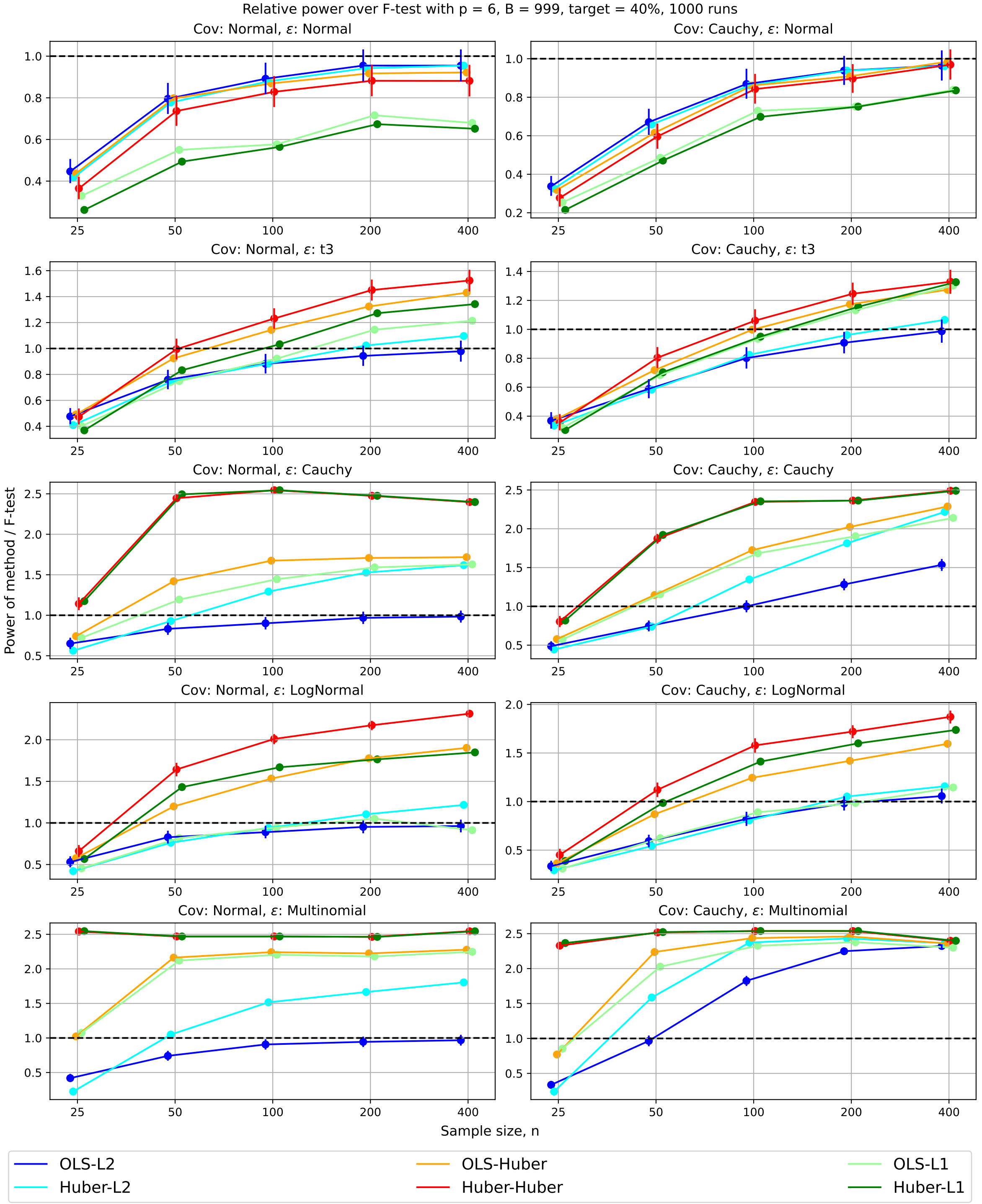

FIGURE D5 Relative power of RobustPALMRT compared to the F-test vs sample size n. In each setting, β is selected to fix the power of the F-test at 40%. Note that the maximum possible ratio of powers is 2.5. There are 1000 replicates with $B = 999$. We show error bars for our method (Huber-Huber) and (OLS-L2)^3, the two of most interest. To improve the visualization of overlapping curves and error bars, we "jittered" curves horizontally, preserving the ratios and the shapes of the curves.

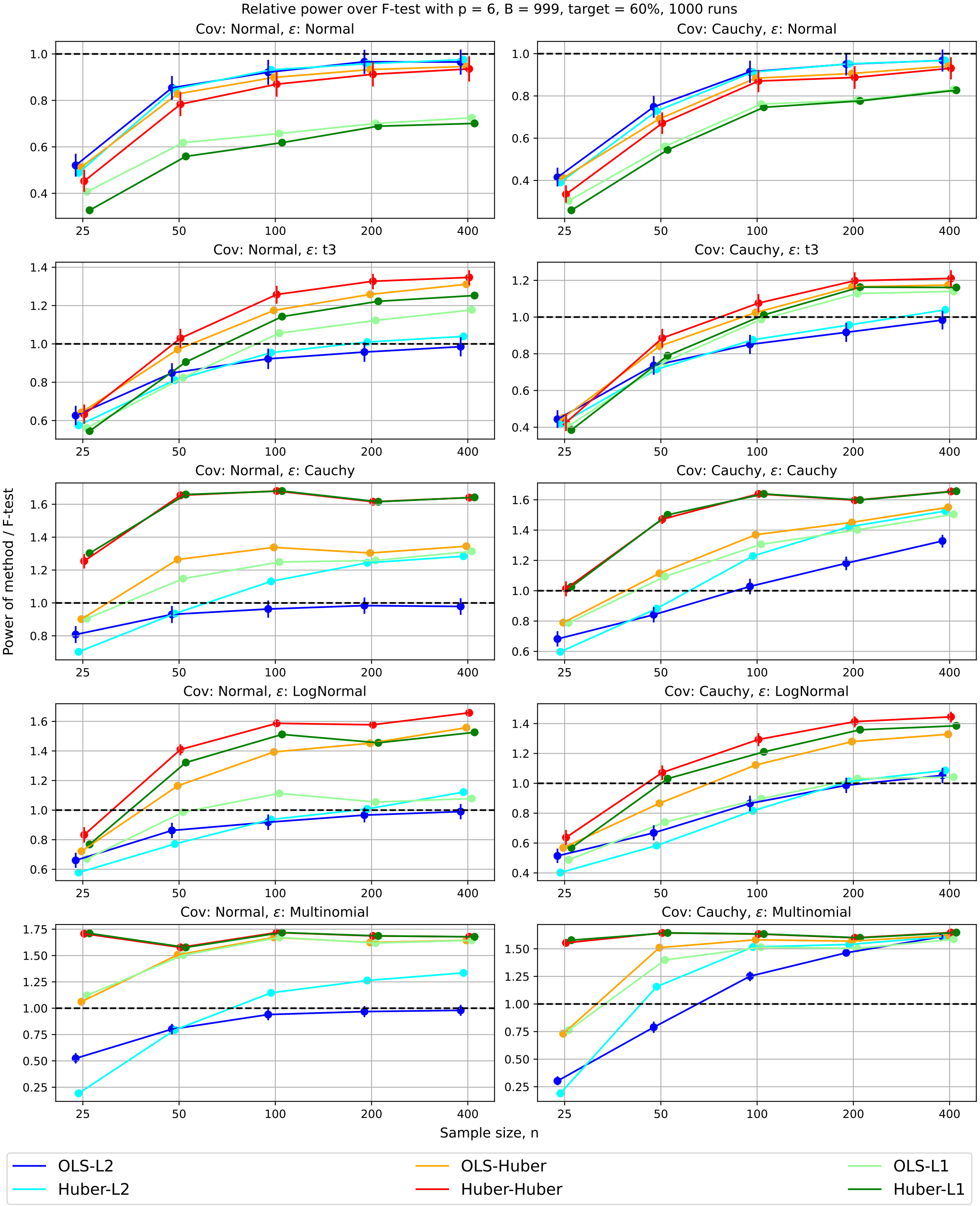

FIGURE D6 Relative power of RobustPALMRT compared to the F-test vs sample size n. In each setting, β is selected to fix the power of the F-test at 60%. Note that the maximum possible ratio of powers is 1.67. There are 1000 replicates with $B = 999$. We show error bars for our method (Huber-Huber) and (OLS-L2)[3], the two of most interest. To improve the visualization of overlapping curves and error bars, we "jittered" curves horizontally, preserving the ratios and the shapes of the curves.

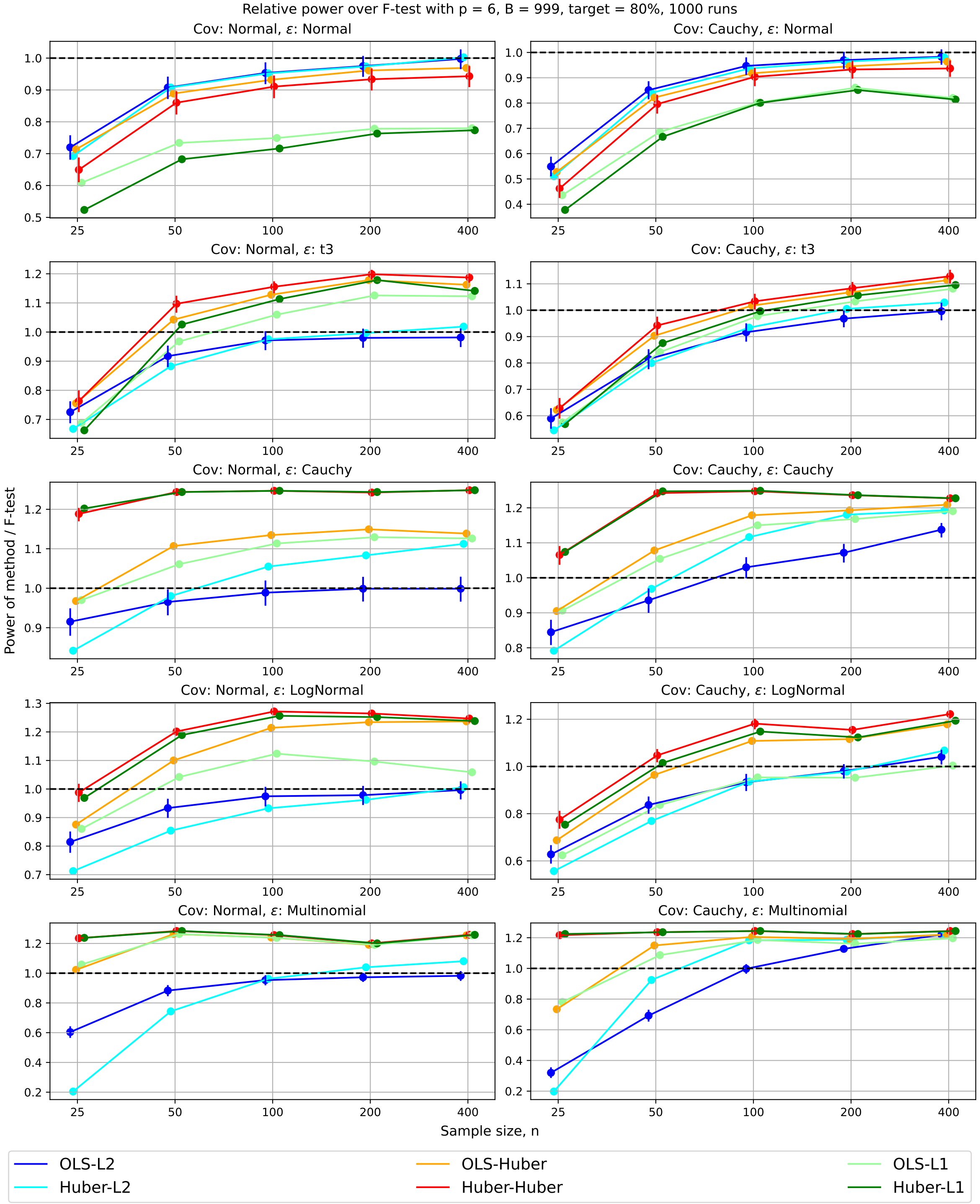

FIGURE D7 Relative power of RobustPALMRT compared to the F-test vs sample size n. In each setting, β is selected to fix the power of the F-test at 80%. Note that the maximum possible ratio of powers is 1.25. There are 1000 replicates with $B = 999$. We show error bars for our method (Huber-Huber) and (OLS-L2)[3], the two of most interest. To improve the visualization of overlapping curves and error bars, we "jittered" curves horizontally, preserving the ratios and the shapes of the curves.

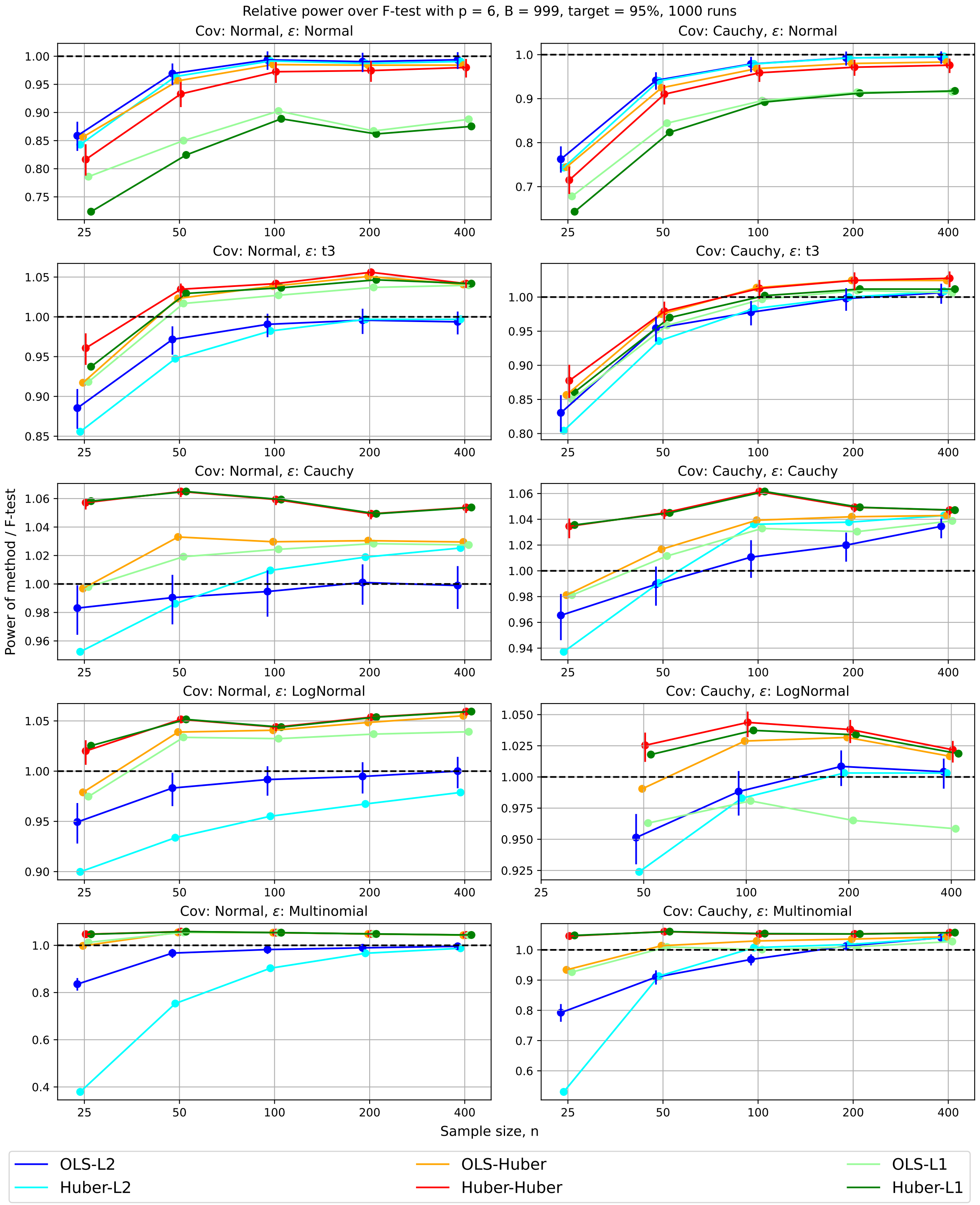

FIGURE D8 Relative power of RobustPALMRT compared to the F-test vs sample size n. In each setting, β is selected to fix the power of the F-test at 95%. Note that the maximum possible ratio of powers is 1.05. There are 1000 replicates with $B = 999$. We show error bars for our method (Huber-Huber) and (OLS-L2)^3, the two of most interest. To improve the visualization of overlapping curves and error bars, we “jittered” curves horizontally, preserving the ratios and the shapes of the curves.

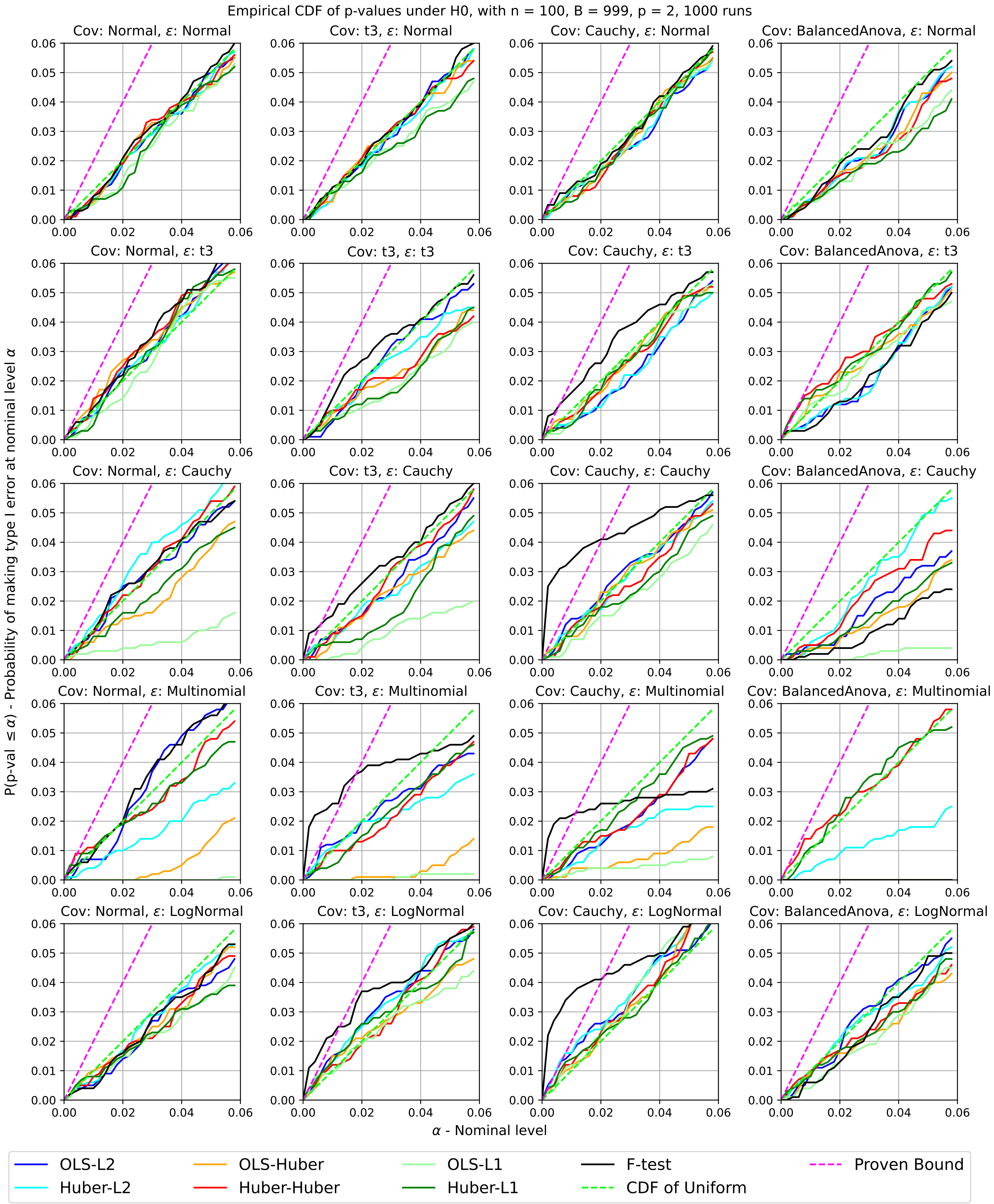

FIGURE D9 Empirical CDF of RobustPALMRT and F-test p-values, with $\beta = 0$, $n = 100$, and $p = 2$. Ideally the empirical CDF (actual p-value) would match the Uniform CDF (nominal p-value; the green dashed line) as closely as possible. Our proofs ensure that the CDF of the RobustPALMRT methods lie below the 2α line (the pink dashed line), but notice that empirically they fall at or below the Uniform CDF.

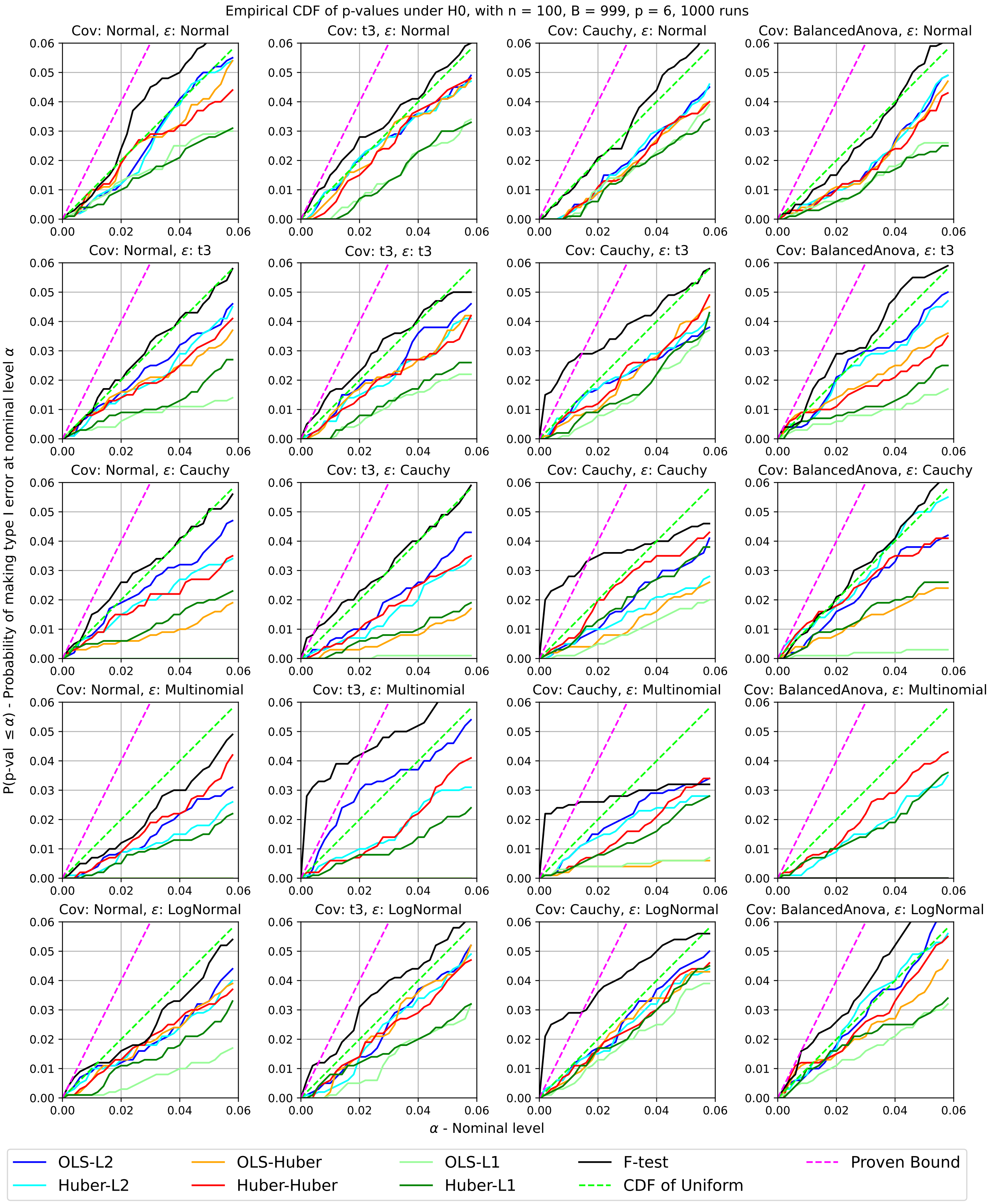

FIGURE D10 Empirical CDF of RobustPALMRT and F-test p-values, with $\beta = 0$, $n = 100$, and $p = 6$. Ideally the empirical CDF (actual p-value) would match the Uniform CDF (nominal p-value; the green dashed line) as closely as possible. Our proofs ensure that the CDF of the RobustPALMRT methods lie below the 2α line (the pink dashed line), but notice that empirically they fall at or below the Uniform CDF.

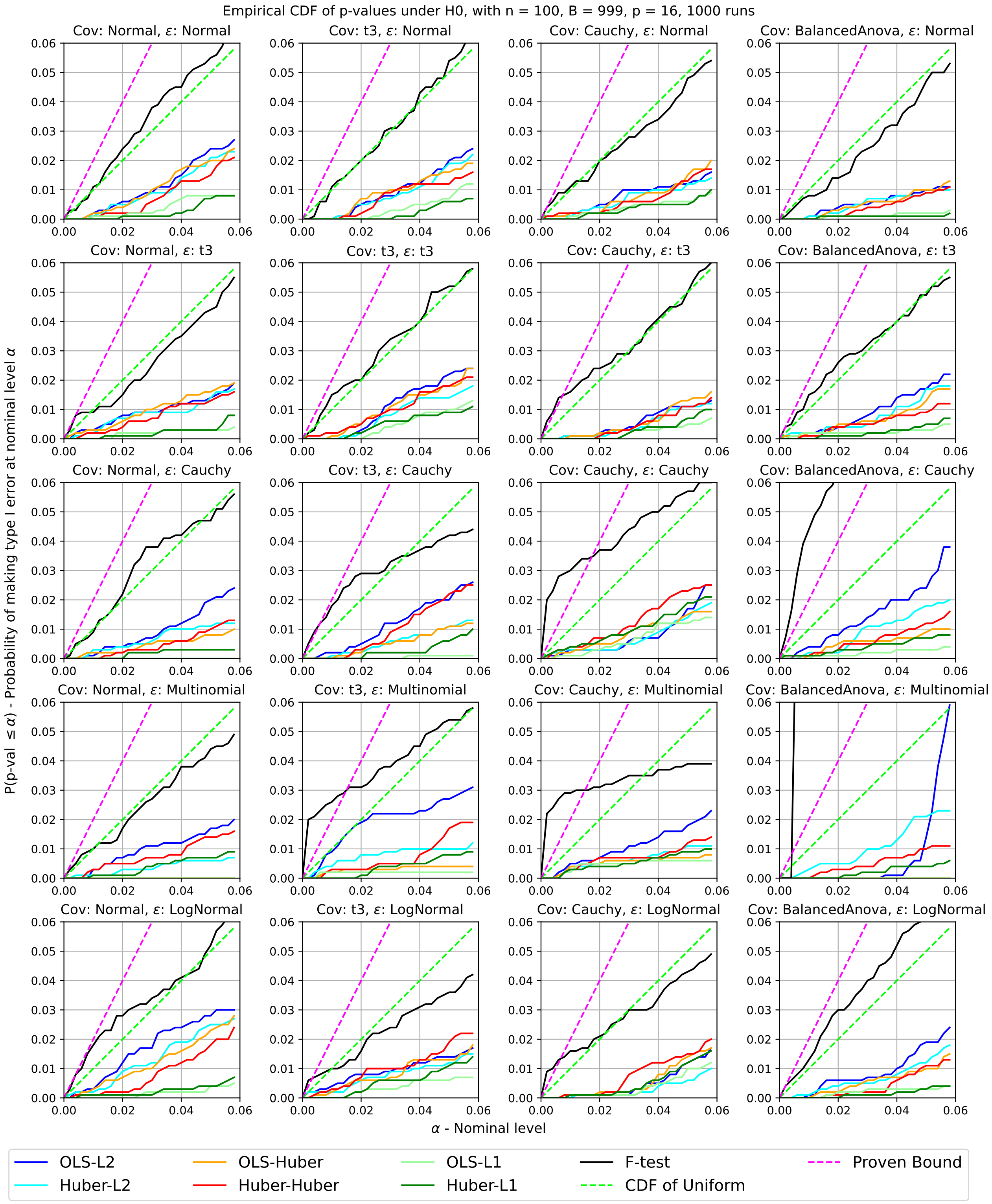

FIGURE D11 Empirical CDF of RobustPALMRT and F-test p-values, with $\beta = 0$, $n = 100$, and $p = 16$. Ideally the empirical CDF (actual p-value) would match the Uniform CDF (nominal p-value; the green dashed line) as closely as possible. Our proofs ensure that the CDF of the RobustPALMRT methods lie below the 2α line (the pink dashed line), but notice that empirically they fall at or below the Uniform CDF.

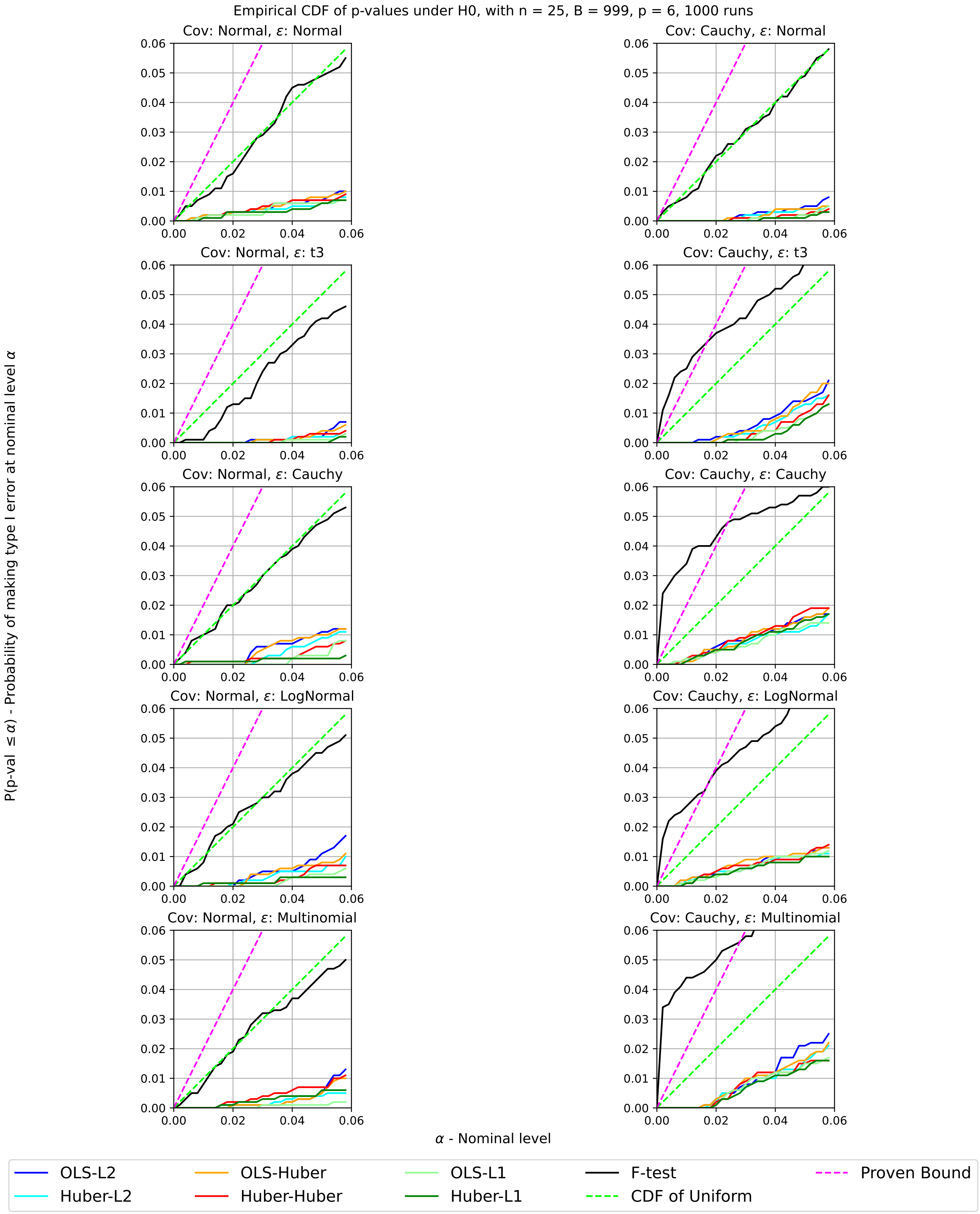

FIGURE D12 Empirical CDF of RobustPALMRT and F-test p-values, with $\beta = 0$, $n = 25$, and $p = 6$. Ideally the empirical CDF would match the Uniform distribution CDF (the green dashed line) as closely as possible. We have proven that the CDF of the RobustPALMRT methods will lie below the 2α line (the pink dashed line), but notice that empirically they fall at or below the Uniform distribution CDF.

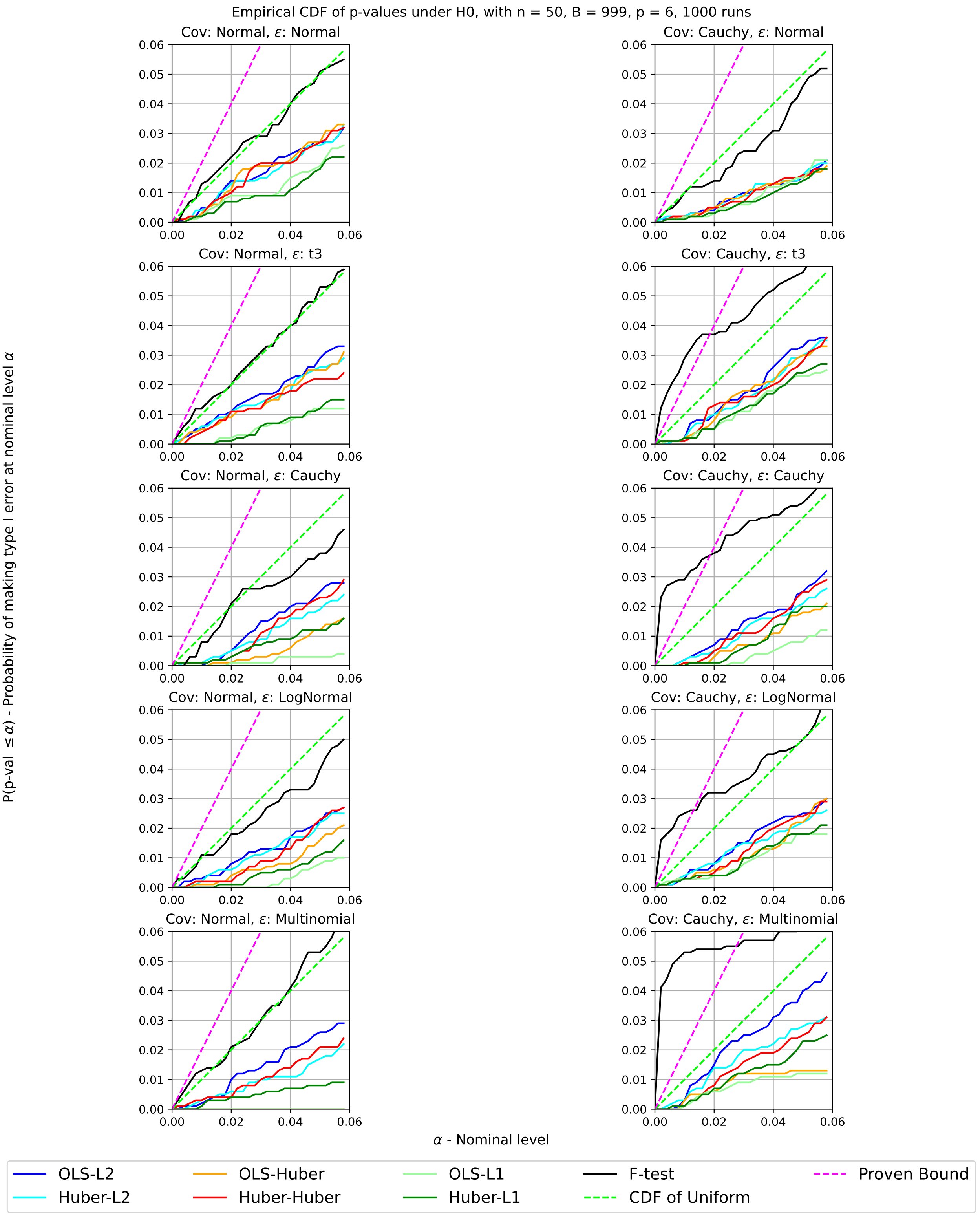

FIGURE D13 Empirical CDF of RobustPALMRT and F-test p-values, with $\beta = 0$, $n = 50$, and $p = 6$. Ideally the empirical CDF (actual p-value) would match the Uniform CDF (nominal p-value; the green dashed line) as closely as possible. Our proofs ensure that the CDF of the RobustPALMRT methods lie below the 2α line (the pink dashed line), but notice that empirically they fall at or below the Uniform CDF.

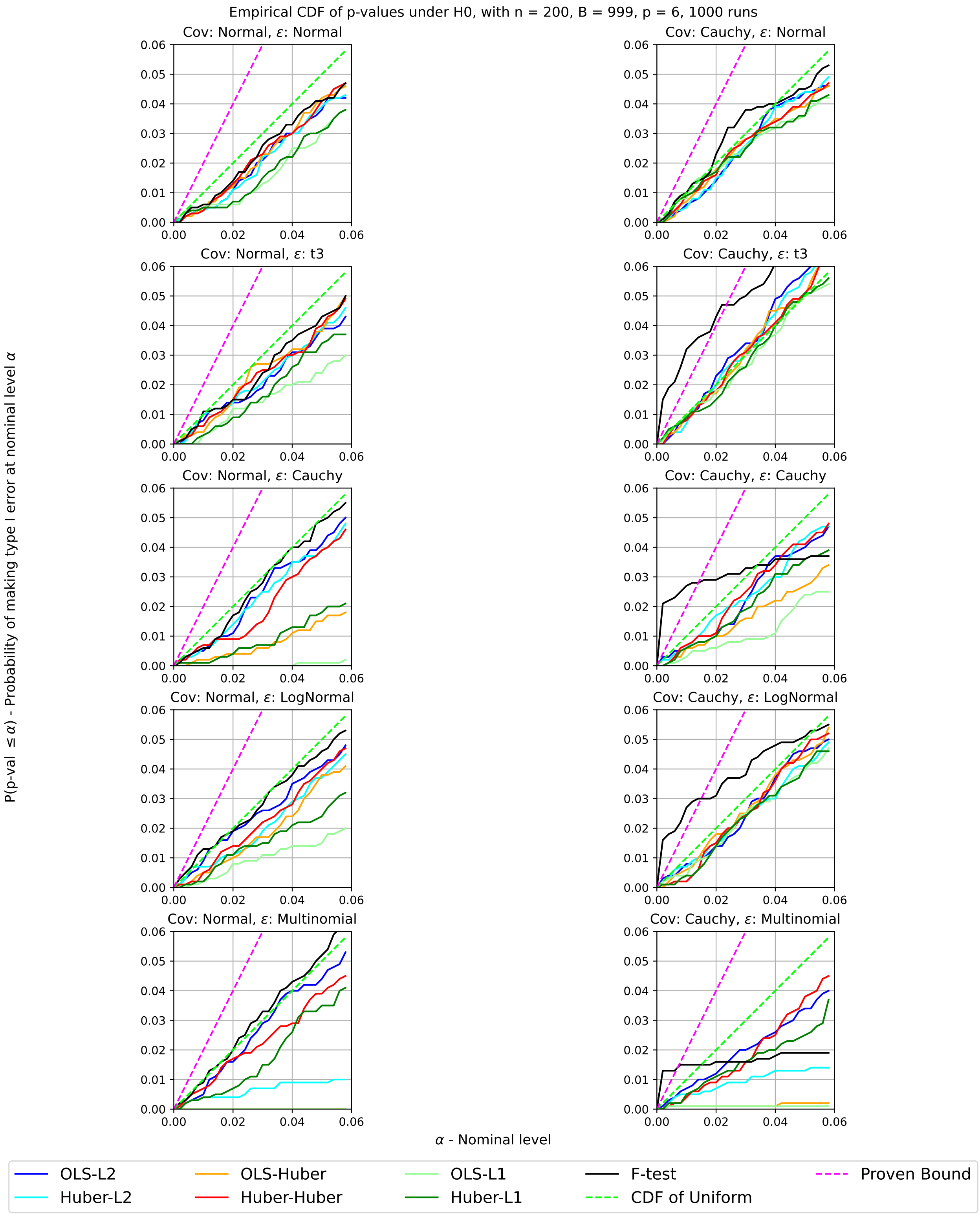

FIGURE D14 Empirical CDF of RobustPALMRT and F-test p-values, with $\beta = 0$, $n = 200$, and $p = 6$. Ideally the empirical CDF (actual p-value) would match the Uniform CDF (nominal p-value; the green dashed line) as closely as possible. Our proofs ensure that the CDF of the RobustPALMRT methods lie below the 2α line (the pink dashed line), but notice that empirically they fall at or below the Uniform CDF.

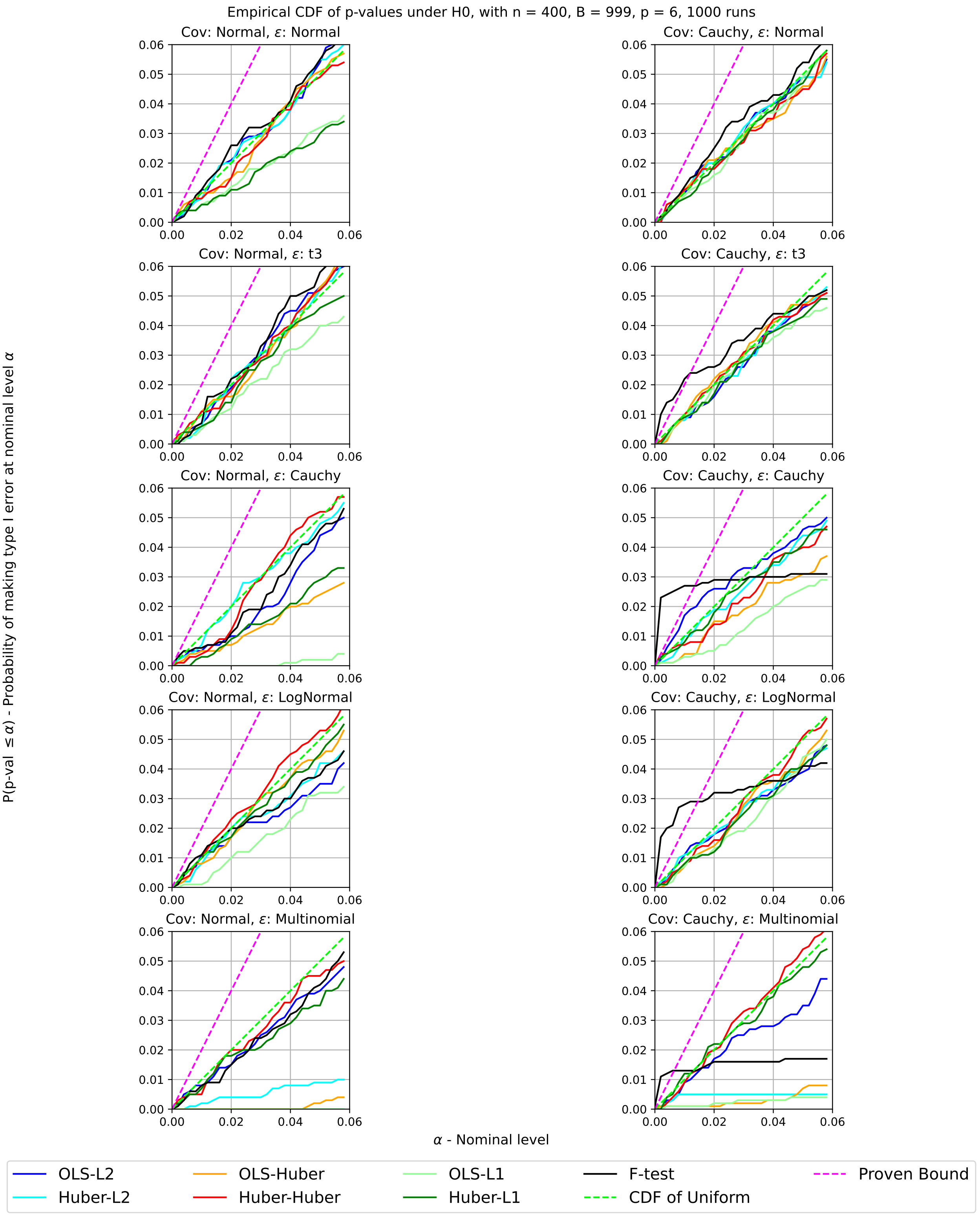

FIGURE D15 Empirical CDF of RobustPALMRT and F-test p-values, with $\beta = 0$, $n = 400$, and $p = 6$. Ideally the empirical CDF (actual p-value) would match the Uniform CDF (nominal p-value; the green dashed line) as closely as possible. Our proofs ensure that the CDF of the RobustPALMRT methods lie below the 2α line (the pink dashed line), but notice that empirically they fall at or below the Uniform CDF.

REFERENCES

1. Venables WN, Ripley BD. *Modern Applied Statistics with S-PLUS*. Springer Science & Business Media, 2013.
2. Virtanen P, Gommers R, Oliphant TE, et al. SciPy 1.0: Fundamental Algorithms for Scientific Computing in Python. *Nature Methods.* 2020;17:261–272. doi: 10.1038/s41592-019-0686-2
3. Guan L. A conformal test of linear models via permutation-augmented regressions. *Ann Stat.* 2024;52(5):2059–2080. doi: 10.1214/24-AOS2421
4. Ramdas A, Barber RF, Candès EJ, Tibshirani RJ. Permutation tests using arbitrary permutation distributions. *Sankhya A.* 2023;85:1156–1177.
5. Vovk V, Wang R. Combining p-values via averaging. *Biometrika.* 2020;107(4):791–808.
6. Barber RF, Candès EJ, Ramdas A, Tibshirani RJ. Conformal prediction beyond exchangeability. *Ann Stat.* 2023;51(2):816–845.
